# Life‐Threatening Fentanyl Patch Poisoning From a Deceased Acquaintance's Leftover Medication: A Case Report

**DOI:** 10.1002/ccr3.73310

**Published:** 2026-08-09

**Authors:** Naoki Yamada, Ko Mitsugi, Akira Iwamura, Hiroshi Morita, Taketsune Kobuchi, Hiroyuki Hayashi

**Affiliations:** ^1^ Department of Emergency Medicine and General Medicine University of Fukui Hospital Fukui Japan; ^2^ School of Medicine, Faculty of Medical Sciences University of Fukui Fukui Japan

**Keywords:** fentanyl patch, leftover medication, naloxone, opioid toxicity, respiratory failure, urine drug screen

## Abstract

Leftover fentanyl patches can cause life‐threatening opioid toxicity in opioid‐naive individuals. In coma with miosis and hypoventilation, management should be guided by the opioid toxidrome, skin examination, patch removal, ventilatory support, and titrated naloxone, even when a routine urine opiate screen is negative.

## 1 Introduction

Transdermal fentanyl patches have been associated with severe and fatal poisoning worldwide, including deaths related to inappropriate use, accidental exposure, and patch misuse [1, 2, 3]. Fentanyl transdermal systems are designed for patients who require continuous opioid analgesia and have sufficient opioid tolerance. Because fentanyl is highly potent and continues to be delivered through the skin, unintended or unsupervised use can rapidly lead to respiratory depression, coma, and death. Safety communications have repeatedly emphasized the need for appropriate storage and disposal of fentanyl patches, including used or no‐longer‐needed patches, because accidental or intentional exposure can be fatal [4, 5].

This case is clinically important not because fentanyl toxicity itself is rare but because a routine emergency department pathway could have been misled by a negative opiate screen, while the key preventive lesson lay in leftover medication access after a patient's death.

## 2 Case History and Examination

A 64 year‐old man with no known opioid prescription and a body weight of 68.6 kg was found convulsing in bed at home. Emergency medical services noted impaired consciousness, agonal respirations, and oxygen saturation in the 70 s. During transport, he developed profound hypoventilation and required assisted ventilation. On arrival at the emergency department, he was deeply comatose with shallow respirations at 6 breaths per minute, pinpoint pupils, blood pressure of 175/105 mmHg, and hypoxemia that improved with high‐flow oxygen and bag‐valve‐mask ventilation.

**FIGURE 1:**
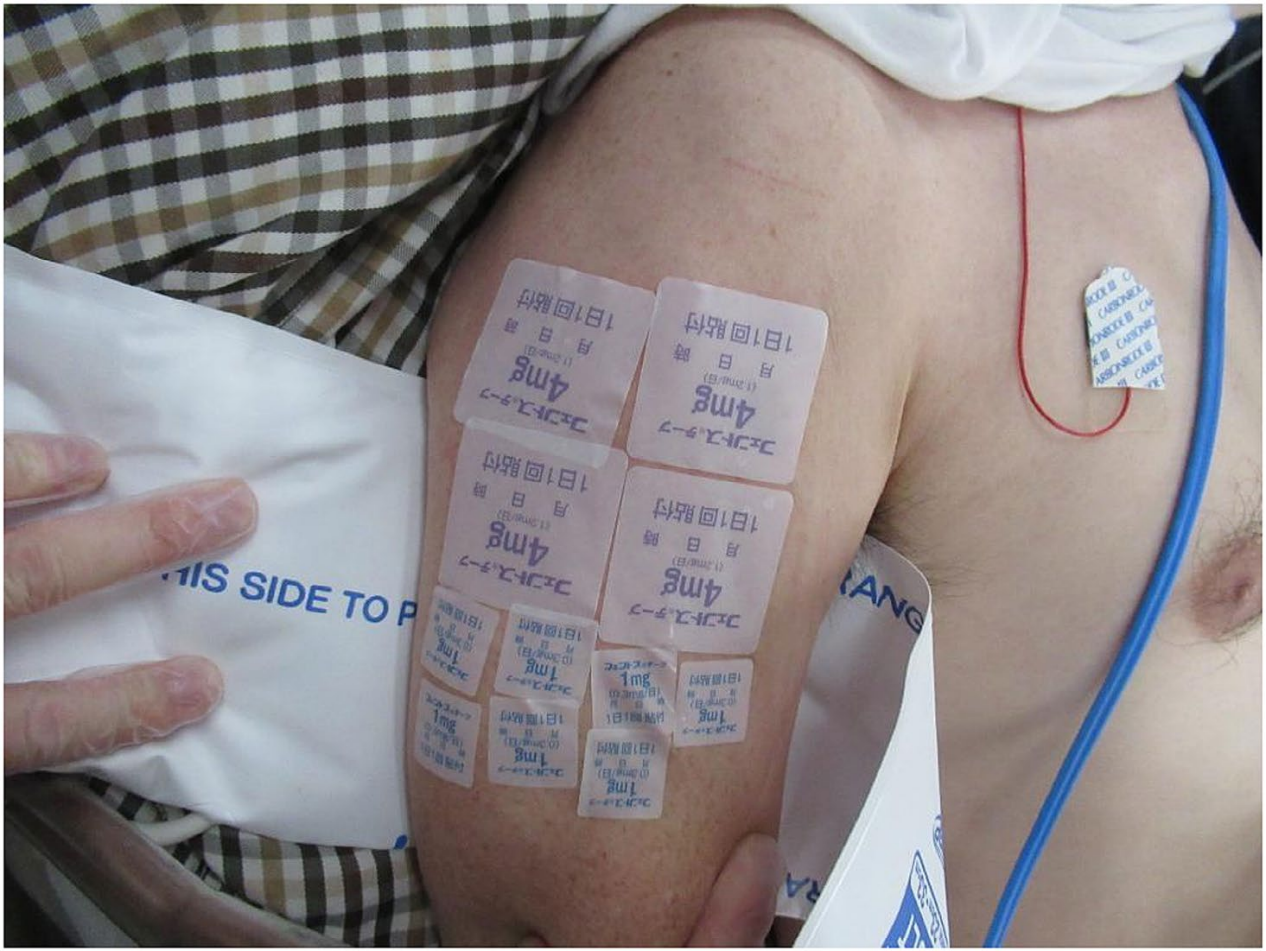
Multiple transdermal fentanyl patches densely applied to the patient's right upper arm. The estimated total labeled fentanyl content was 23 mg, consisting of four 4 mg patches and seven 1 mg patches.

During removal of his clothing, multiple transdermal patches were identified densely applied to the right upper arm (Figure 1). The estimated total labeled fentanyl content was 23 mg, consisting of four 4 mg patches and seven 1 mg patches. The patient had no prescription record for fentanyl or other opioids. Arterial blood gas analysis on high‐flow oxygen showed pH 7.25 and PaCO_2_ 53 mmHg, consistent with acute hypercapnic respiratory failure. Initial blood tests did not reveal severe metabolic derangement sufficient to explain the coma. Hepatic enzyme levels were within the reference range, and renal function was mildly reduced, with an estimated glomerular filtration rate of 60.8 mL/min/1.73 m^2^.

## 3 Investigations and Treatment

Because the patient had severe hypoventilation and depressed consciousness, endotracheal intubation was performed for airway protection and ventilatory support. Whole‐body computed tomography showed no intracranial hemorrhage, major trauma, or other alternative cause of coma or respiratory failure. The fentanyl patches were removed during the initial emergency department care.

**FIGURE 2:**
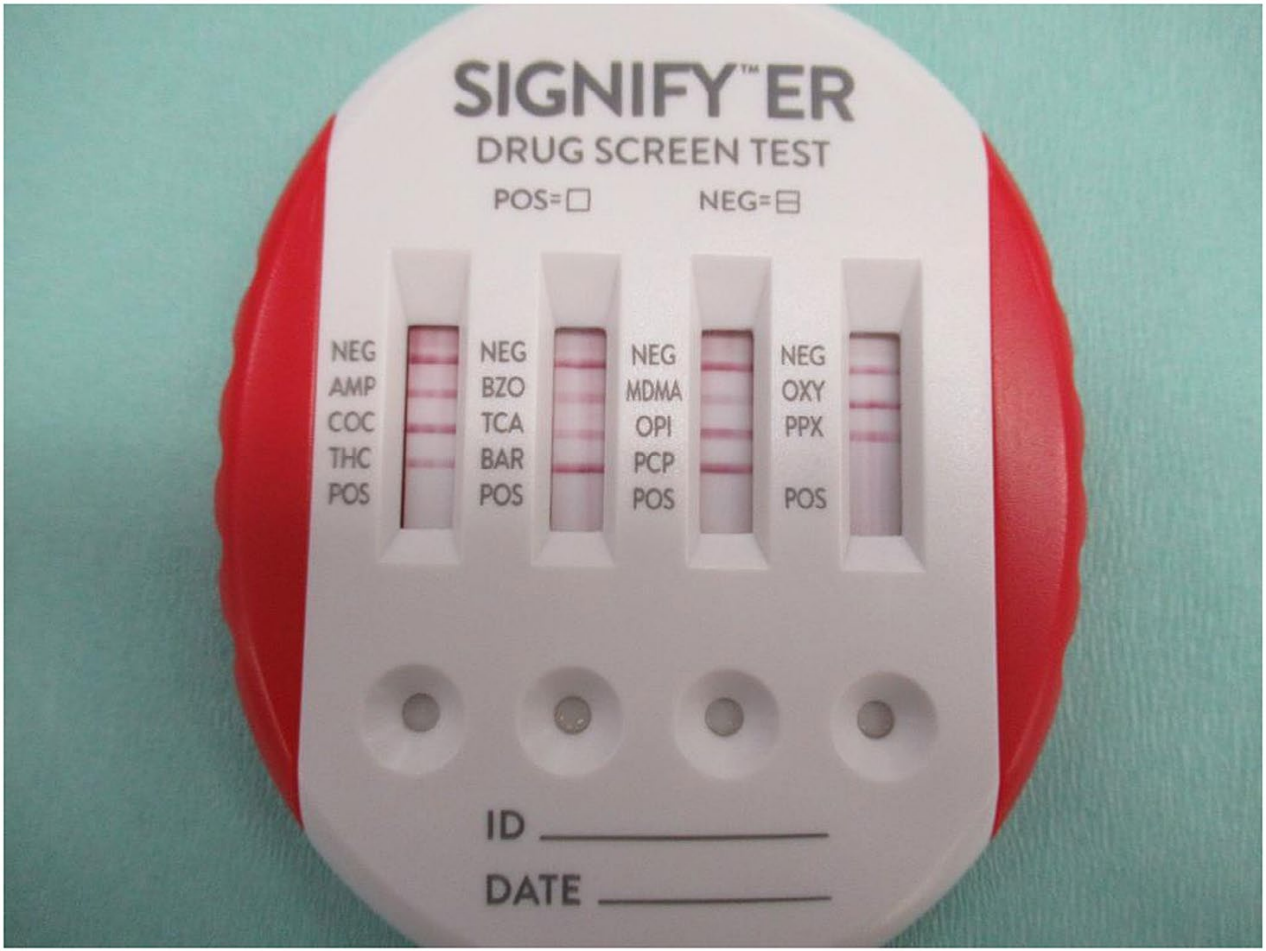
Qualitative multi‐panel urine drug screen obtained approximately 2 h after arrival. The routine “OPI” panel was negative despite clinical findings strongly consistent with fentanyl toxicity. The result was interpreted as a limitation of a non‐fentanyl‐specific immunoassay.

A qualitative multi‐panel urine drug screen (Signify ER; Sysmex, Kobe, Japan) was obtained approximately 2 h after arrival. The result was negative for the routine “OPI” panel despite the strongly suggestive opioid toxidrome and the visible fentanyl patches (Figure 2). The manufacturer information for this assay indicates that it primarily targets morphine‐type opioids and does not reliably detect fentanyl, even when high concentrations are added to urine. Therefore, the negative result was interpreted as a limitation of the routine non‐fentanyl‐specific immunoassay rather than evidence against fentanyl toxicity.

Intravenous naloxone 0.2 mg was administered during ongoing ventilatory support when fentanyl poisoning remained the leading diagnosis. The patient was admitted to the intensive care unit for close monitoring, sedation management, and mechanical ventilation. Because transdermal fentanyl may continue to be absorbed after patch removal, respiratory monitoring was continued after the patches had been removed.

## 4 Outcome and Follow‐Up

The patient's level of consciousness and spontaneous ventilation gradually improved. On hospital day 2, a spontaneous breathing trial was attempted but failed because of recurrent apnea and persistent hypercapnia. On hospital day 3, spontaneous breathing was sustained, and he was successfully extubated. He was transferred from the intensive care unit to the general ward later that day and was discharged home on hospital day 7 without neurologic sequelae.

Further history from the patient and family clarified the source of exposure. The patches had been found among the belongings of a deceased acquaintance who had received fentanyl for cancer pain. The patient had chronic shoulder pain and had applied the patches himself, believing that they were ordinary analgesic plasters and underestimating their potency. He stated that he would not have used them had he understood the risk.

## 5 Discussion

This case conveys four practice messages. First, leftover fentanyl patches can become a source of life‐threatening opioid poisoning after a patient's death. Warnings about accidental fentanyl patch exposure often focus on children, transfer from a patient's skin, or ingestion of discarded patches [4, 5]. This case shows that adults without opioid tolerance may also be harmed when leftover patches are mistaken for benign topical analgesics. For prescribers, pharmacists, and inpatient teams, counseling should not stop at correct application. It should also include explicit instructions not to share patches, not to keep unused patches after they are no longer needed, and to return or dispose of them according to local regulations.

Second, this case illustrates how differences in fentanyl patch labeling can obscure the magnitude of opioid exposure. In Japan, some fentanyl tapes are labeled by the nominal patch dose in milligrams and are replaced every 24 h, whereas many transdermal fentanyl systems used in Europe are labeled by the delivery rate in micrograms per hour and are replaced every 72 h. The 23 mg of fentanyl tape applied in this patient corresponded approximately to an estimated mean fentanyl absorption of 6.9 mg/day, or 287.5 μg/h, based on Japanese conversion data (Table 1) [6]. Given his body weight of 68.6 kg, this was approximately 4.2 μg/kg/h. This standardized expression helps international readers interpret the severity of exposure and allows cautious comparison with previous reports, including the fatal case reported by Voigt, in which a 75 μg/h fentanyl patch was mistaken for a heat plaster [7]. Although direct comparison is limited by differences in patch duration and formulation, the estimated delivery rate in our case underscores the potential lethality of multiple Japanese fentanyl tapes in an opioid‐naïve adult.

**TABLE 1:** Approximate conversion of Japanese Fentanyl tape dose to estimated Fentanyl absorption rate.

Japanese tape dose	Replacement interval	Estimated mean fentanyl absorption	Relevance
1 mg	24 h	0.3 mg/day = 12.5 μg/h	Seven patches = 87.5 μg/h
4 mg	24 h	1.2 mg/day = 50 μg/h	Four patches = 200 μg/h
23 mg total	24 h	6.9 mg/day = 287.5 μg/h	Total estimated exposure

*Note:* Values are approximate estimates derived from Japanese package insert and conversion data for once‐daily fentanyl tape [6]. The milligram strength indicates the nominal tape dose and does not mean that the entire fentanyl content is delivered over 24 h. The fentanyl tapes used in this case were once‐daily transdermal formulations replaced every 24 h, whereas many fentanyl patch systems used in Europe are labeled by delivery rate and replaced every 72 h. Actual systemic exposure may vary according to formulation, skin temperature, skin perfusion, application site, body composition, hepatic and renal function, and opioid tolerance.

Third, emergency management should be guided by the opioid toxidrome rather than by a routine urine opiate screen. The combination of coma, miosis, and hypoventilation strongly suggested opioid toxicity. However, many routine hospital immunoassays are calibrated to morphine‐like compounds and may not detect fentanyl or its analogs [8]. A negative non‐fentanyl‐specific opiate screen therefore cannot exclude fentanyl poisoning. In the present case, the physical finding of multiple patches was more diagnostically important than the urine test. This is particularly relevant when patches are hidden on the back, trunk, or under clothing, or when the history is unavailable.

Fourth, treatment should integrate patch removal, ventilatory support, and titrated naloxone. Naloxone should be titrated to restore adequate ventilation and protective reflexes rather than to achieve complete arousal [9]. In patients with profound hypoventilation, airway management and antidotal therapy are complementary rather than competing interventions. This case also illustrates that transdermal fentanyl toxicity may persist after patch removal, so observation should continue even after initial improvement.

The main limitation of this report is that serum fentanyl or norfentanyl concentrations were not measured. The diagnosis was based on the opioid toxidrome, direct visualization of multiple fentanyl patches, exclusion of alternative causes, and clinical improvement after supportive care and naloxone. Nevertheless, this pattern is sufficient for the intended clinical message: routine urine screening should not override bedside findings, and safe management of leftover fentanyl patches is a preventable safety issue.

## 6 Conclusion

Leftover fentanyl patches may be misunderstood as ordinary analgesic plasters and can cause life‐threatening respiratory depression in opioid‐naive adults. In patients with coma, miosis, and hypoventilation, clinicians should perform meticulous skin examination, remove transdermal drug systems, provide ventilatory support, and use titrated naloxone when opioid toxicity is suspected, regardless of routine urine opiate screen results.

## Author Contributions

**Naoki Yamada:** conceptualization, writing – original draft, writing – review and editing. **Ko Mitsugi:** writing – review and editing, resources. **Akira Iwamura:** writing – review and editing. **Hiroshi Morita:** writing – review and editing. **Taketsune Kobuchi:** writing – review and editing. **Hiroyuki Hayashi:** writing – review and editing.

## Funding

The authors have nothing to report.

## Ethics Statement

According to the policies of University of Fukui Hospital, single‐patient case reports do not require formal Institutional Review Board review.

## Consent

Written informed consent for publication, including the clinical images, was obtained from the patient.

## Conflicts of Interest

The authors declare no conflicts of interest.

## Data Availability

The data that support the findings of this study are available on request from the corresponding author. The data are not publicly available due to privacy or ethical restrictions.
